# The Role of Levetiracetam in Treatment of Seizures in Brain Tumor Patients

**DOI:** 10.3389/fneur.2013.00153

**Published:** 2013-10-07

**Authors:** Ekokobe Fonkem, Paul Bricker, Diana Mungall, Jose Aceves, Eromata Ebwe, Wei Tang, Batool Kirmani

**Affiliations:** ^1^The Brain Tumor Center, Scott & White Healthcare, Temple, TX, USA; ^2^Texas A&M Health Science Center College of Medicine, Temple, TX, USA; ^3^Epilepsy Center, Scott and White Neuroscience Institute, Temple, TX, USA

**Keywords:** intravenous levetiracetam, seizures, brain tumor patients, antiepileptic drugs, neurologic complications

## Abstract

Levetiracetam, trade name Keppra, is a new second generation antiepileptic drug that is being increasingly used in brain tumor patients. In patients suffering with brain tumors, seizures are one of the leading neurologic complications being seen in more than 30% of patients. Unlike other antiepileptic drugs, levetiracetam is proposed to bind to a synaptic vesicle protein inhibiting calcium release. Brain tumor patients are frequently on chemotherapy or other drugs that induce cytochrome P450, causing significant drug interactions. However, levetiracetam does not induce the P450 system and does not exhibit any relevant drug interactions. Intravenous delivery is as bioavailable as the oral medication allowing it to be used in emergency situations. Levetiracetam is an attractive option for brain tumor patients suffering from seizures, but also can be used prophylactically in patients with brain tumors, or patients undergoing neurological surgery. Emerging studies have also demonstrated that levetiracetam can increase the sensitivity of Glioblastoma tumors to the chemotherapy drug temozolomide. Levetiracetam is a safe alternative to conventional antiepileptic drugs and an emerging tool for brain tumor patients combating seizures.

## Introduction

Individuals with brain tumors represent a very challenging patient population for clinicians. Not only must clinicians deal with and treat the primary tumor, but they must also manage the numerous accompanying sequelae. Seizures are commonly seen in brain tumor patients, with reports of 30% or more depending on the type of tumor (1). In fact, an epileptic seizure is the presenting symptom of a tumor in 30–50% of patients, and 10–30% of those patients will go on to develop recurrent seizures over the course of the disease (2, 3). The presence of seizures and convulsions has been shown to add substantial morbidity to these patients (4).

## Role of Anticonvulsants in Acute Seizure Management and Seizure Prophylaxis in Brain Tumor Patients

Current consensus states that all patients with brain tumors should be treated with antiepileptic drugs. Currently, the first-line medications including phenytoin, carbamazepine, or valproate all have demonstrated major side-effects and dangerous drug interactions with commonly used chemotherapy and tumor medications. The most common side-effects seen with these highly used antiepileptic medications include cognitive impairment, bone-marrow suppression, liver dysfunction, and dermatological symptoms (5). Reinforcing the complexity of care in these cases are several studies demonstrating that side-effects are more frequent in patients with brain tumors compared with the overall epileptic population (3, 6). Tsai et al. conducted a retrospective chart review to assess the effect of valproic acid (VPA) on the outcome of patients with glioblastoma multiforme (GBM). The electronic medical records were queried from January 2004 to December 2006 and 102 patients newly diagnosed with GBM were found. Those patients were followed until January 2010 and over that time 87 patients died due to disease progression, 7 patients lived and were followed between 43.4 and 61.0 months, and 8 patients were lost to follow up. VPA was administered at a starting dose of 400 mg every 8 h in the form of a sodium salt. It was then adjusted as needed to serum level or seizure activity. Patients were analyzed in two groups: an “early” treatment group in which the patients began VPA treatment within 2 weeks of initial diagnosis and the “late” treatment group where the patients began treatment with VPA more than 2 weeks after initial diagnosis.

Seven patients on VPA therapy provided tissue samples from a second resection procedure for analysis of histone acetylation. When these samples were analyzed, there was an increase in acetylation in a small subset of patients. Eighty-five of 102 patients (83.3%) underwent radiotherapy, 61/102 patients (59.8%) underwent chemotherapy, and all patients had a neurosurgical procedure with the most common being total excision (47.1%). Thirty-three of 102 patients had VPA therapy and of those 16 patients (58.5 serum concentration) started therapy less than a week after diagnosis and 17 patients (52.4 serum concentration). The early treatment group had an average serum VPA level of 58.46 μg/ml (34.1–72.6) and the late group was 52.37 μg/ml (36.3–73.2). When the early treatment group was evaluated with univariate analysis there appeared to be a survival benefit conferred (*P* = 0.035). However, when a stratified analysis according to chemotherapy was used, VPA therapy was not associated with a statistically significant difference (*P* = 0.315) in overall survival. Mild nausea and thrombocytopenia were the most commonly reported side effect (11/33 and 11/33, respectively). The authors concluded that VPA therapy did not affect patient survival significantly (7).

Drug interactions between antiepileptic drugs and commonly used tumor therapies can lead to inadequate control of the seizures or sub-therapeutic treatment of the tumor. Toxic effects have also been noted, leading to unnecessary morbidity. Many of the common antiepileptic drugs induce cytochrome P450 enzymes which cause faster metabolism and lower concentrations within plasma (5). Corticosteroids and many other chemotherapy drugs exhibit decreased effectiveness in the presence of enzyme activating antiepileptic drugs (8, 9). The reciprocal relationship is also seen as there are chemotherapeutic agents that induce enzymes of the P450 system, lowering concentrations of antiepileptic drugs (3, 10).

Within the past 10 years, several new antiepileptic drugs including lamotrigine, levetiracetam, oxcarbazepine, lacosamide, topiramate, and zonisamide have emerged that are without clinically relevant drug interactions (11–13). Data are limited for these newer agents and the main limitation in use is lack of intravenous formulation. Maschio et al. studied the effects of 12 months of oxcarbazepine (OXC) monotherapy on seizure control in patients with brain tumor-related epilepsy (BTRE) in a prospective, observational study. Eleven women and 14 men (mean age 49.7 years, range 25–75) with BTRE were enrolled between September 2007 and January 2009. Enrolled subjects had a histological diagnosis of meningiomas, primary grade I gliomas, low grade gliomas, anaplastic gliomas, or multiform glioblastoma. Epileptic patients were eligible if they had simple or complex seizures with or without secondary generalization, if they had greater than or equal to two seizures per month on no AEDs before referral to their center, or patients that had been treated at the maximum tolerated dosage with other AEDs. Twenty-four patients received monotherapy with OXC of which 17 patients were *de novo* and 7 patients rare on monotherapy with one AED. A baseline was established at the first visit of seizure frequency, neurological examination, Zung self-depression rating scale (ZSDRS), adverse events profile, and patients were given a seizure diary. During week one OXC began and any prior AED was tapered gradually over the first 3 weeks. The OXC dose started at 300 mg/day and was titrated every 4 days by 300 mg/day up to 2,100 mg/day over 4 weeks.

The mean follow up duration was 7.1 months (range 1–12 months). Five patients died as a result of tumor progression and 10 patients dropped out due to severe side-effects (*N* = 6), uncontrolled seizures (*N* = 3), and a lung complication (*N* = 1). Six patients (24%) had severe side-effects due to rash (*N* = 4), confusion (*N* = 1), and dizziness (*N* = 1) and one patient (4%) had a mild rash. Four patients had no systemic therapy, 1 had radiotherapy, 4 had chemotherapy, and 16 had both radiotherapy and chemotherapy. Patients received a mean OXC dosage of 1,230 mg/day (range 600–2,100 mg/day). Of the 10 patients who completed the 12 months of follow up, there was a significant seizure reduction (*P* = 0.005) in the mean weekly seizure number from 2.62 ± 6.35 at baseline to 0.13 ± 0.37 in the final follow up. There was a significant difference in the seizure freedom rate (*P* = 0.002) by the McNemar’s test between the baseline and final follow up intent to treat population. Using logistic regression analysis, the authors found that the efficacy of OXC in seizure control was not affected by chemotherapy and radiotherapy (*P* = 0.658). The ZSDRS showed significantly increased mood in patients in the final follow up (*P* = 0.011). Thus, the researchers concluded that OXC was efficacious in controlling seizures. However, lack of intravenous formulation limits its use in acute cases. The other agent used is lamotrigine which is also not available in intravenous formulation (14).

Meyer et al. conducted a prospective study in order to determine the protein binding and distribution of LTG in serum, brain tissue, and brain tumor in three female and eight male subjects with brain tumors. From 1994 to 1996, 11 patients had neurosurgical operations for benign tumors (*N* = 2) and malignant tumors (*N* = 9). The subjects were aged 33–68 years (mean 56 years). Patients were enrolled if in the days immediately preceding neurosurgery they showed signs of seizures or if their preoperative EEG showed symptoms of epilepsy. For intraoperative and postoperative seizure control, subjects were administered LTG with PHT in seven patients or LTG with PHT and CBZ in three patients. Patients received a mean of 54.4 days of preoperative treatment (range 1 day–17 months) with LTG, and 2 h preoperatively were given a dose of 100–200 mg/day in addition to PHT or PHT and CBZ. After administration, serial blood samples were taken from 0.5 to 12.5 h, as well as an intraoperative blood sample and intraoperative removed tumor tissue (*N* = 6). HPLC was used to assay LTG concentrations and an ultrafiltration system was used to determine plasma binding of LTG.

The LTG concentration at the time of tumor sectioning was on average 3.7 μg/ml (range 1.1–9.8) in the serum, an average of 6.8 μg/g (range 1.0–14.9) in the brain tissue, and an average of 4.4 μg/g (range 2.0–8.3) in the tumor tissue. The resultant brain/serum ratio was 2.8 and the tumor/serum ratio was 1.9. The protein binding of LTG was determined to be a lipophile AED. The researchers concluded that LTG penetrates well into brain and tumor tissue and had a moderately high protein binding (15). It has been shown that pregabalin has been used as adjunctive therapy in this subgroup of patients although limited data are available, which is the case with most other newer agents (16).

Lacosamide is one of the newest agents which has the advantage of being available in both an oral and intravenous formulation. Few studies reported the efficacy as an adjunctive agent, and data are limited at this time.

Saria et al. conducted a retrospective chart review to study the use of lacosamide in patients with brain tumors. Seventy patients with primary brain tumors who received lacosamide for seizure control were identified by reviewing the medical records of five United States medical centers with brain tumor programs. Primary tumors included glioblastoma (40%), grade II gliomas (36%), and were followed by grade III anaplastic astrocytomas, anaplastic/atypical meningiomas, anaplastic ependymomas, and pleomorphic xanthoastrocytomas. Seventy-eight percent of patients (*N* = 55) had partial seizures and 17% had of patients (*N* = 12) had generalized seizures. Subjects had a mean age of 51 years of age. Eighty-four percent of patients had chemotherapy (*N* = 59) and 81% had radiation therapy (*N* = 57). The majority of patients had a surgical procedure performed, including 63% had a craniotomy, 23% had a biopsy, and 14% had both. Most patients (83%) were on an additional AED as well as lacosamide, which was most commonly levetiracetam. The cause for the addition of lacosamide therapy was most commonly recurrent seizures (74%) or toxicity from another AED (23%).

A decrease in seizure frequency was found in 46 patients (66%) and seizure control remained unchanged in 21 patients (30%). A greater than 50% decrease in seizure frequency was achieved in 38 patients (83%). No medication toxicities were reported in 77% of patients (*N* = 54) receiving lacosamide. The most common toxicity reported was fatigue noted by four subjects (6%). The researchers concluded that in patients with brain tumors lacosamide was well tolerated and an active add-on AED (17).

Maschio et al. also conducted a case series of 14 patients with BTRE that had not obtained adequate seizure control on other AED treatment. Patients were consecutively recruited if they had at least one seizure per month prior to study recruitment. Lacosamide was the first-fifth add-on AED therapy and was started at 100 mg/day and titrated weekly by 100 mg/day to a maximum dosage of 400 mg/day.

Follow up occurred in subjects at a mean of 5.4 months (range < 1–10 months). During this period, nine patients died from tumor progression, no patients’ underwent radiation therapy, and nine patients underwent chemotherapy. The mean lacosamide dosage was 332.1 mg/day (range 100–400 mg/day). One subject dropped out due to dizziness and blurred vision. The mean seizure frequency at baseline was 15.4 per month and at follow up decreased to 1.9 per month. Subjects had a median seizure reduction percentage of 79.8%. Five patients (35.7%) had a greater than 50% seizure reduction and six patients (42.9%) were seizure free. One patient (7.1%) had an unmodified seizure frequency. There was a statistically significant difference in the mean monthly seizure frequency from baseline to follow up (*P* = 0.022). The authors concluded that lacosamide is a valid alternative add-on AED in patients with BTRE (18) have had seizures.

## Role of Levetiracetam in Acute Seizure Management and Seizure Prophylaxis in Brain Tumor Patients

In this article we chose to focus on the use of levetiracetam. In our experience we have witnessed better efficacy and there seems to be more evidence within the literature supporting levetiracetam in terms of seizure management. An important point to make here is that there are many antiepileptic drug choices, but very little comparative effectiveness data to help physicians decide the best AED to use in the innumerably variable clinical scenarios. This paper attempts to make a contribution and demonstrate what we feel is a good drug choice for managing seizures in the unique population of patients with brain tumors. Levetiracetam is very different from the more commonly used antiepileptic drugs (19, 20). Its action is believed to involve neuronal binding to synaptic vesicle protein 2A (SV2A). Binding to this protein somehow acts as an inhibitor of synaptic vesicle exocytosis (21) decreasing presynaptic neurotransmitter release (21). The mechanisms of action for the more commonly used antiepileptic drugs including benzodiazepines and barbiturates affect gamma-aminobutyric acid potentiation, calcium channels, or sodium channels (22).

Levetiracetam exhibits a relative bioavailability of 100% following both oral (23) and intravenous administration. Levetiracetam, given intravenously, is considered bioequivalent to oral tablets and is well tolerated (12). Being able to give this drug intravenously is an extremely attractive trait which makes treatment in emergency or perioperative situations a possibility. Perhaps more importantly is the fact that levetiracetam is not extensively metabolized by the cytochrome P450 system (23). This is in extreme contrast to the most commonly used antiepileptic drugs. Levetiracetam has not been shown to cause any induction or inhibition of the other important cytochrome P450 enzymes including uridine diphosphate-glucuronyl-transferase or epoxide hydroxylase (23). Levetiracetam has repeatedly been shown to exhibit a low potential for clinically relevant pharmacokinetics both with other antiepileptic drugs or drugs that could possibly be used to treat brain tumors (13, 23). Studies have demonstrated no or very few side-effects with levetiracetam treatment in patients with brain tumors who also received antineoplastic agents (7, 24–26). A few of the larger studies were able to report the occasional occurrence of somnolence with initial doses of levetiracetam (27).

## Seizure Management with Levetiracetam

Over the past 10 years, there have been several studies that looked at both the effectiveness and safety of using levetiracetam in brain tumor patients. Levetiracetam, as both an adjunct therapy and a monotherapy, has shown a complete seizure control rate of between 47.4 and 100% (7, 25–27). Reductions in seizures by more than 50% were recorded in a majority of articles within the literature. These numbers were extremely variable ranging from 29 to 100%. However most of these studies were conducted with limited numbers of patients and were mostly retrospective analyses (6, 11, 24, 25, 28–32). The two largest studies and the ones most likely indicative of what clinicians will see in their patients are from a 2005 *Neurology* supplement by Stevens et al. (31) and a report from Rosati et al. (27) in 2010. The study in *Neurology* reviewed the medical charts of 278 patients with varying brain tumors treated with levetiracetam over a 36-month period. They witnessed a greater than 50% reduction in seizure activity in over 60% of their patients. The second largest study, by Rosati et al., involved 176 patients in a prospective study over a 3-year period (27). In this study, 91% of patients were seizure free with a monotherapy of levetiracetam. Forty-nine of the patients (60%) experienced fast and long-lasting seizure control with initial doses of 1,500–3,000 mg/day. In 23 patients (31.5%) an increase in the dosage up to 3,000–4,000 mg/day was necessary because of sub-therapeutic drug levels. The authors experienced no relevant laboratory abnormalities (27). Levetiracetam has demonstrated good potential however; larger cohorts over more extended periods of time would be useful.

While levetiracetam has demonstrated good potential as an adjunct therapy as well as monotherapy, several alternative uses for levetiracetam are currently being explored such as refractory status epilepticus.

Traditional seizure medications have proven woefully ineffective with over half of brain tumor patients continuing to have seizures despite treatment (32). Status epilepticus is seen in up to 26% of these cases (6) with the overall mortality rate reaching 30–40% (33). First-line treatment for status epilepticus in this patient population is a combination of benzodiazepines and phenytoin (34), which has an efficacy rate of 60–70%. Refractory status epilepticus requires an additional anesthetic drug such as propofol or midazolam to induce an iatrogenic coma (35). Recent data suggest that an alternative combination of phenytoin and levetiracetam has proven to be a very effective cocktail that does not subject the patient to mechanical ventilation or sedation (35).

Swisher et al. conducted a retrospective chart review of electronic medical records for all patients with a diagnosis of primary or metastatic brain tumor who presented with complex partial refractory status epilepticus and received Trifecta (intravenous PHT, LEV, and oral PGB). All patients were >18 years of age and presented between January 2006 and December 2009. Study subjects ages ranged from 25 to 84 with an average age of 56.9. There was a prior history of seizures in 70% of patients. GBM occurred in 52% of patients and was the most common tumor type, yet the tumor locations of all subjects tumors was highly variable. Ninety-one percent of patients had underwent resection for their brain tumor. Thirty-nine percent of patients received radiation, 39% received chemotherapy, and within 1 month of RSE onset 52% underwent a biopsy or brain tumor resection. PHT or LEV was used as first-line therapy in all patients and pregabalin was typically used as second or third-line therapy. The median dosage of LEV was 3,000 mg/day and the median dosage of PGB was 375 mg/day.

Ninety-one percent of patients were already on one AED when RSE was diagnosed, and it was statistically significant (*P* = 0.03) that more patients in the responder group were on an AED at baseline (100 vs. 71%). The average PHT blood level in the Trifecta responder patients was 18.9 at the time of SE cessation. After the administration of Trifecta 30% of patients’ (7/23) seizure frequencies were unchanged. After the administration of Trifecta, status epilepticus ceased in 70% of patients (16/23). On average, 24 h after the addition of the third AED status epilepticus was aborted. There was a zero mortality rate in the responder group and only one patient in the responder group required intubation. There were no adverse reactions to Trifecta reported. The authors concluded that in patients with brain tumors presenting in RSE that Trifecta use is highly effective and safe (16).

## Surgical Prophylaxis

Patients with brain tumors often must undergo neurological surgery for resection or biopsy, which in and of itself can increase risk of seizures. So controlling seizures in the perioperative phase of brain tumor management is an important consideration (3, 36).

Bahr et al. performed an open-label, prospective, single-arm study investigating the use of LEV for perioperative seizure control in patients with suspected primary brain tumors undergoing neurosurgery (e.g., biopsy or resection) (36). Inclusion criteria included age >18 years, neuroradiological imaging suspected primary brain tumor, and planned biopsy or resection neurosurgical procedure. Patients were excluded if in the seven preoperative days any AED other than LEV was used or if there was a known LEV allergy or previous severe side-effects to LEV. Between January 2008 and June 2009, 25 of 30 study patients were enrolled and treated for 4 weeks before and 4 weeks after a neurosurgery procedure with oral and IV LEV. During this time patients had four scheduled visits for monitoring and data collection. Of the 27 patients who underwent surgery, 22 were diagnosed with a primary brain tumor, 3 with meningioma, 2 with brain metastasis, and 1 with abscess. Subjects were started on an oral dose of LEV 500 mg twice daily and titrated to 1,000 mg twice daily after 72 h. The dose was further increased if the patient was at an increased risk of seizures or had seizures in this period. IV LEV was given immediately and for 36 h postoperatively before being transitioned back to oral LEV. Three patients did not have the planned procedure and two patients were lost to follow up.

In the pre-surgery phase (defined as 3 days–4 weeks prior to surgery), 100% of patients were seizure free after the initiation of LEV therapy. In the 48 h post surgery phase and early follow up phase (defined as 48 h–4 weeks post surgery), the seizure free rates were 88 and 84%, respectively. Three patients failed LEV treatment, even after the dose was titrated up to 3,000 mg/day. There were no serious adverse events reported with LEV treatment. The authors concluded that oral and IV LEV for perioperative seizure control was feasible and safe in patients with tumor-related seizures (36). However, to date only four studies have been able to demonstrate the effectiveness of levetiracetam in the perioperative phase (32–35). Zachenhofer et al. (37) retrospectively studied 78 patients with brain tumors who received between 1,000 and 3,000 mg of LEV perioperatively. After a mean follow up time of 10.5 months, 91% of the patients were seizure free. This study demonstrated a very low seizure frequency of 2.5% in the early postoperative period. These studies propose both the feasibility and safety of intravenous levetiracetam in the perioperative treatment, but more long term trials are needed.

## Increasing Chemotherapy Sensitivity

Levetiracetam’s role in increasing chemotherapy sensitivity is a fascinating new field of interest. Some antiepileptic drugs have actually shown that they can inhibit histone deacetylase activity within the tumor. Histone deacetylase inhibitors can modulate temozolomide activity by modulating methylguanine-DNA-methyltransferase (MGMT) expression (37) thereby allowing for increased temozolomide efficacy. Levetiracetam has in fact been shown to increase the transcription of histone deacetylase 1 (HDAC1) which ultimately silences MGMT (38). Levetiracetam has also been shown to have a neuroprotective role via free-radical scavenging activity (39), reducing inflammation and neuronal death (40). All of these attributes may lead to the ability of levetiracetam to prevent radiochemotherapy-caused nerve damage. Levetiracetam may in fact increase temozolomide-induced cytotoxicity in patients with GBM who do express the MGMT protein while also experiencing little adverse side-effects (41, 42). This could be a very exciting area of research as GBM tumors are so common.

## Conclusion

Brain tumor patients require a multidisciplinary approach involving the use of chemotherapy, radiation, possible surgery, and in many cases antiepileptic drugs. The first-line treatments for these patients have numerous drug interactions to be weary of when using them in addition to anticancer drugs. Increased side-effects have led to renewed interest in antiepileptic drugs that do not induce cytochrome P450 pathways. Levetiracetam, a drug unique in both its mechanism of action and in the way that it does not cause the drug interactions of the first-line drugs, is among the newer antiepileptic drugs. Several studies have demonstrated the effectiveness of levetiracetam in the role of monotherapy as well as adjunct therapy to other antiepileptic drugs. Levetiracetam has also sparked the interest of clinicians for its role in surgical prophylaxis, for refractory status epilepticus, and the ability to increase chemotherapy sensitivity. Some of these attributes have more supporting evidence than others, but levetiracetam does warrant more testing and could very well be a promising drug in the fight to control epilepsy in brain tumor patients.

## Conflict of Interest Statement

The authors declare that the research was conducted in the absence of any commercial or financial relationships that could be construed as a potential conflict of interest.
